# Supplementary material for: Association of Blast Exposure in Military Breaching with Intestinal Permeability Blood Biomarkers Associated with Leaky Gut
Source: Int J Mol Sci. 2024 Mar 21;25(6):3549. doi: 10.3390/ijms25063549 (PMC10971443; doi:10.3390/ijms25063549)
Supplement: Supplementary file 1 [file ijms-25-03549-s001.zip › ijms-2834508-supplementary.pdf]

	Pre-post		Pre-followup	
	Spearman r	p	Spearman r	p
**Alpha diversity**	-0.0684	0.7813	-0.3175	0.185
**Zonulin**	0.2966	0.247	0.0686	0.7947
**LBP**	0.4923	**0.0398**	**0.7564**	**0.0004**
**Claudin3**	0.2962	0.232	-0.226	0.3656
**I-FABP**	0.1702	0.4979	0.1765	0.4821

Table S1. Correlations between alpha-diversity and IP biomarker change from pre to post and followup with cumulative blast exposure measured in peak pressure (psi) * time (ms),with corresponding significance reported.

symptom	project	case/total	pre-post_mean_sd_case	pre-post_mean_sd_control	pre-post_cohen	pre-follow_mean_sd_case	pre-follow_mean_sd_control	pre-follow_cohen
headache	alpha	16/30	0.0427 (0.6055)	0.1564 (0.5153)	-0.201 (small)	0.2721 (0.6364)	0.124 (0.6917)	0.223 (small)
	zonulin	14/22	1.1974 (1.6473)	1.3275 (2.0654)	-0.072 (negligible)	0.954 (1.6076)	1.8361 (1.8286)	-0.523 (medium)
	lbp	15/24	-1.8617 (3.3232)	-1.0881 (2.7425)	-0.248 (small)	0.8061 (3.5807)	1.9689 (2.9081)	-0.347 (small)
	claudin 3	15/24	-3.4096 (3.2147)	-2.714 (3.274)	-0.215 (small)	-2.2727 (2.8112)	-1.0025 (3.9167)	-0.39 (small)
	ifabp	15/24	9.285 (86.8141)	-3.7389 (50.757)	0.172 (negligible)	51.7331 (77.5267)	99.8699 (70.7096)	-0.641 (medium)
longthink	alpha	12/30	-0.0061 (0.5332)	0.1637 (0.5797)	-0.302 (small)	0.1641 (0.7646)	0.229 (0.594)	-0.097 (negligible)

	zonulin	11/22	1.7371 (1.437)	0.7523 (1.9809)	0.569 (medium)	0.8922 (1.5514)	1.6573 (1.8351)	-0.45 (small)
	lbp	11/24	-1.5367 (2.7482)	-1.6011 (3.4472)	0.02 (negligible)	0.1899 (3.2837)	2.1324 (3.2204)	-0.598 (medium)
	claudin 3	11/24	-3.4471 (4.0118)	-2.8963 (2.4222)	-0.17 (negligible)	-2.5503 (3.0328)	-1.1584 (3.4024)	-0.43 (small)
	ifabp	11/24	7.4382 (88.775)	1.8311 (63.2986)	0.074 (negligible)	33.0698 (68.2858)	100.8507 (72.5572)	-0.959 (large)
concentration	alpha	8/30	0.2981 (0.6308)	0.0222 (0.5263)	0.498 (small)	0.2819 (0.8835)	0.1743 (0.5743)	0.162 (negligible)
	zonulin	5/22	1.8162 (2.0463)	1.0766 (1.7022)	0.416 (small)	1.6501 (1.63)	1.1644 (1.7576)	0.28 (small)
	lbp	5/24	-1.9488 (2.5422)	-1.4723 (3.2609)	-0.152 (negligible)	-0.5383 (3.1656)	1.7107 (3.2885)	-0.689 (medium)
	claudin 3	5/24	-4.5792 (4.6941)	-2.7724 (2.709)	-0.571 (medium)	-3.3161 (2.9033)	-1.3964 (3.2841)	-0.597 (medium)
	ifabp	5/24	49.2902 (86.4709)	-7.4119 (68.4927)	0.786 (medium)	50.5683 (37.1332)	74.8413 (84.7645)	-0.31 (small)
dizziness	alpha	9/30	-0.0526 (0.8091)	0.1593 (0.42)	-0.379 (small)	0.2588 (0.8186)	0.1791 (0.594)	0.12 (negligible)
	zonulin	7/22	1.4128 (1.8046)	1.1663 (1.8006)	0.137 (negligible)	1.4496 (1.6216)	1.1932 (1.7903)	0.147 (negligible)
	lbp	7/24	-1.7 (2.9141)	-1.5187 (3.231)	-0.058 (negligible)	0.5315 (2.6784)	1.5347 (3.5945)	-0.298 (small)
	claudin 3	7/24	-4.3759 (4.0453)	-2.6435 (2.7396)	-0.55 (medium)	-2.4964 (2.8123)	-1.5081 (3.4473)	-0.301 (small)
	ifabp	7/24	35.8016 (73.0228)	-8.5286 (73.0326)	0.607 (medium)	38.3837 (45.3616)	82.7141 (84.8115)	-0.582 (medium)

Table S2. Association of changes in self-reported symptoms vs. changes in alpha-diversity and IP biomarkers, shown as effect sizes

measured by Cohen's d. For each elevated symptom self-reported, participants were assigned to either of two groups i.e., increasing vs. non-increasing by comparing the symptom scales reported at pre vs. post and pre vs. follow-up per subject. Then the increasing vs. the non-increasing groups by symptom were compared for differences in alpha-diversity and levels of IP biomarkers (separately) across timepoints (specifically post - pre and follow-up - pre). Mean and standard deviation of alpha-diversity and biomarkers for each group are reported along with the effect size.
